# Loss of choroid plexus‐derived insulin‐like growth factor 2 (IGF2) leads to hyposmia, while retaining post‐partum mood resilience in mice

**DOI:** 10.1111/jne.70058

**Published:** 2025-06-03

**Authors:** Hollian R. Phillipps, Eleni C. R. Hackwell, Ionel Sandovici, Miguel Constância, David R. Grattan

**Affiliations:** ^1^ Centre for Neuroendocrinology and Department of Anatomy School of Biomedical Sciences, University of Otago Dunedin New Zealand; ^2^ University of Cambridge Metabolic Research Laboratories and MRC Metabolic Diseases Unit Institute of Metabolic Science, Addenbrookes Hospital Cambridge UK; ^3^ Department of Obstetrics and Gynaecology and National Institute for Health Research Cambridge Biomedical Research Centre Cambridge UK; ^4^ Centre for Trophoblast Research, Department of Physiology, Development and Neuroscience University of Cambridge Cambridge UK; ^5^ Maurice Wilkins Centre for Molecular Biodiscovery University of Auckland Auckland New Zealand

**Keywords:** choroid plexus, insulin‐like growth factor 2, olfaction, prolactin

## Abstract

During the post‐partum period, new mothers are vulnerable to mood disorders. In adults, impairments in neurogenesis commonly associate with anxiety and depressive behaviors. Insulin‐like growth factor 2 (IGF2) is expressed in the choroid plexus (CP) within the subventricular zone (SVZ) neurogenic niche, and global loss of IGF2 leads to increased anxiety. Previously, we have shown that *Igf2* expression in CP tissue increases 6‐fold during lactation but returns to baseline on suppression of prolactin present in lactation, suggesting it is induced by high levels of prolactin. To gain more insight into the role of prolactin‐induced *Igf2* expression in the CP, we have measured IGF2 levels in cerebrospinal fluid across reproductive states and developed mice in which *Igf2* is conditionally removed from the CP. Using CP‐derived IGF2 knockout mouse models, we have measured *Prlr* expression in CP tissue, SVZ mitogenesis, olfaction, and anxiety‐like behavior using an elevated plus maze (EPM) and light/dark transition test (LDTT). Interestingly, we observed a reduction in *Prlr* expression in CP tissue in one of our *Igf2* knockout mouse models, suggesting *Igf2* may also act upstream to regulate *Prlr* expression in CP tissue. No changes were detected in SVZ proliferation rates between *Igf2* knockout and controls. Using a buried food test (BFT), however, we show mice with conditional loss of *Igf2* in the CP take longer to find a buried fruit loop as compared to controls, indicating olfaction deficits. Overall anxiety levels, however, were comparable between knockout and controls in the EPM and LDTT. Together, our findings reveal loss of CP‐derived IGF2 leads to hyposmia in the absence of detectable changes to SVZ mitogenesis. We propose that CP‐derived IGF2 may be acting directly in the olfactory bulb to elicit changes to improve olfaction, which may become particularly important during the post‐partum period to facilitate mother–pup interactions.

## Graphical abstract

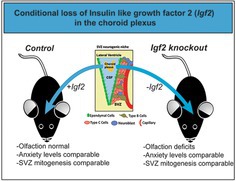

## 1 INTRODUCTION

Despite collective recognition for the choroid plexus (CP) as the primary source of cerebrospinal fluid (CSF), it has been surprisingly understudied.^1^, ^2^ Since this tissue is in close association with the subventricular zone (SVZ), a known proliferative site for neural stem cells (NSCs) in adults, it is considered a key component of the SVZ neurogenic niche.^3^ Hence, it is important to understand what proteins are being released by the CP and elucidate their functional consequences to gain a more comprehensive understanding of how proteins in CSF support brain function. Insulin‐like growth factor 2 (IGF2), a mitogenic polypeptide, is produced at high levels by the CP and secreted into CSF. Circulating levels of IGF2 diminish with age^4^ and in adults, expression is predominantly restricted to regions in the brain, including the CP, endothelial cells, leptomeninges, and NSCs in the subgranular zone of the hippocampus.^5^, ^6^, ^7^ Interestingly, both *Igf2* alleles are transcriptionally active in the CP, unlike other brain regions where *Igf2* is imprinted, being expressed solely by the paternal allele.^5^, ^8^ Additionally, decreased NSC activation in the SVZ is observed in *Igf2* mutant mice that contain only one transcriptionally active allele (either maternal or paternal), indicating that gene dosage in the CP is functionally important.^5^ This suggests that IGF2 may play a particularly important role in this tissue. Recent work has suggested that CSF‐derived IGF2 may act in a paracrine manner to maintain and/or regulate differentiation of the NSC pool within the SVZ neurogenic niche.^5^, ^9^

Across reproductive states the rate of SVZ mitogenesis is variable in mice, with proliferation peaks being observed in early pregnancy and again in early lactation,^10^ driven by changes in prolactin secretion from the anterior pituitary gland.^10^, ^11^ Interestingly, prolactin also regulates *Igf2* expression in the CP during lactation,^6^ a period synonymous with high prolactin levels.^12^ Previous work investigating the functional consequences of prolactin‐induced SVZ mitogenesis showed that blocking prolactin secretion using the dopamine D2 agonist bromocriptine during early pregnancy caused consequential deficits in maternal care and increased anxiety‐like behavior post‐partum.^10^, ^11^ The specific mechanisms responsible for these adaptative changes in the brain are largely unknown. We hypothesized that prolactin‐induced IGF2 from the CP may be inducing neurogenesis in specialized brain niches, thereby contributing to adaptative neuroplastic changes across different physiological states.^13^

**FIGURE 1:**
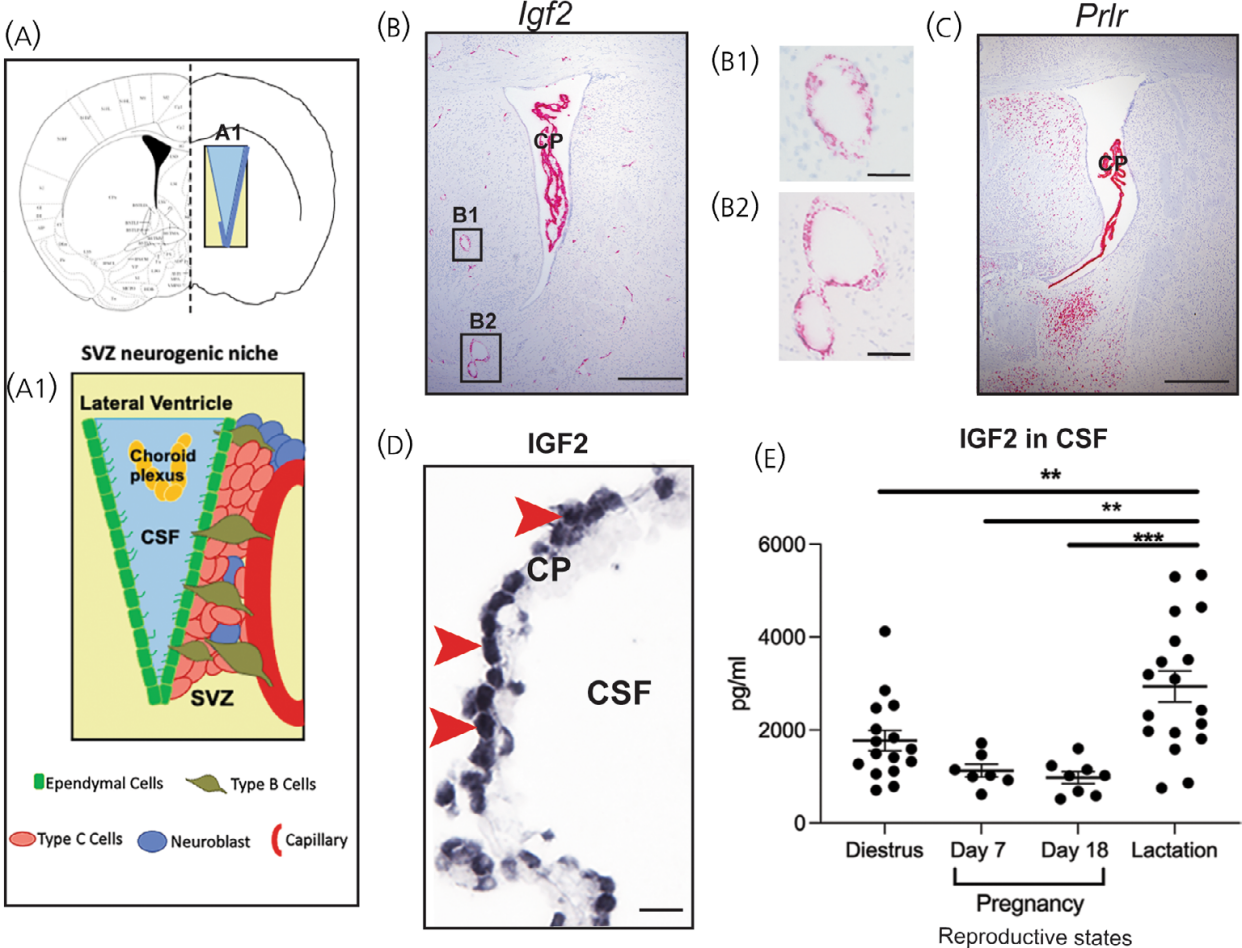
IGF2 expression at gene and protein levels in the SVZ neurogenic niche. (A) Coronal view of mouse brain with location of SVZ neurogenic zone indicated at A1; which is shown as an enlarged schematic below highlighting all components of the SVZ neurogenic niche lying within and alongside the lateral ventricle. *Igf2* (B) and *Prlr* (C) expression in the SVZ neurogenic niche detected by RNAscope assay (red: Positive hybridization). Note the high levels of expression in epithelial cells of the CP. Boxed areas indicate examples of positive hybridization for *Igf2* in endothelial cells shown as higher‐powered images (B1 and B2). (D) Immunohistochemistry for IGF2 expression, immunopositive cells shown in black (examples indicated by red arrows). Note IGF2 is expressed in the majority of epithelial cells in the CP. (E) Graph showing levels of IGF2 in CSF across reproductive states. Data are shown as individual values with mean +/− SEM indicated; **p < .01 and ***p < .001 by one‐way ANOVA with Tukey post hoc test. SVZ, Subventricular Zone; CP, Choroid Plexus; CSF, Cerebrospinal Fluid. Scale bars 200 μm (B and C), 50 μm (B1, B2, D).

The SVZ neurogenic niche is a highly proliferative area, where NSCs continuously self‐renew under both physiological and pathological stimuli.^14^ It consists of the CP, CSF within the ventricles, ependymal cells lining the lateral ventricle, and a number of NSC types, including type B and C cells within the SVZ (Figure 1A).^15^ NSCs in this region can differentiate into neuroblasts (immature neurons), which can then migrate along the rostral migratory stream to the olfactory bulb and become incorporated into either the granular or glomerular layers as functional interneurons.^15^ Hence, the olfactory bulb is highly neuroplastic due to continuous neurogenesis occurring in adulthood. The function of new neurons integrating into existing circuitry in the olfactory bulb is still poorly understood; however, previous work indicates that adult neurogenesis in this region may play a role in odor discrimination and odor memory development.^16^, ^17^ In the hippocampus, NSCs in the subgranular zone (SGZ) migrate to the granule cell layer within the dentate gyrus and integrate into existing circuitry as glutamatergic granule neurons.^18^ These neurons play a role in learning and memory.^19^ IGF2 from the CP is unlikely to have an effect on neurogenesis in this region based on the fact that autocrine regulation of IGF2 occurs in NSCs residing in the SGZ^5^ and a previous study involving intracerebroventricular infusion of fibroblast and epidermal growth factors in rats expanded the NSC population in the SVZ but showed no effect on NSC proliferation rates in the hippocampus.^20^

Previous work has shown that global removal of *Igf2* in adulthood leads to a reduction in NSC turnover in the SVZ, increased recruitment of immature neurons to the olfactory bulb accompanied by heightened anxiety‐like behavior and hyposmia in mice.^21^ Conditional removal of *Igf2* from blood vessels also leads to reduced proliferation of the SVZ NSC pool and a consequential decrease in immature neurons in the olfactory bulb.^5^ Here, we aimed to extend this knowledge by determining the function of IGF2 specifically derived from the CP. First, we characterized the IGF2 secretion profile in CSF across reproductive states and then investigated whether prolactin‐induced *Igf2* derived from the CP is required for SVZ neurogenesis in lactation to maintain post‐partum mood and behavior. Finally, we measured *Prlr* expression levels in CP tissue from *Igf2* knockout mice to determine whether reciprocal regulation of *Igf2* and *Prlr* occurs in CP tissue.

## 2 MATERIALS AND METHODS

### 2.1 Ethics statement

All animal experimental procedures were approved by the University of Otago Animal Ethics Committee in accordance with the New Zealand Animal Welfare Act 1999 (AUP 20/93).

### 2.2 Animals

Experimental mice were sourced from the Biomedical Resource unit (University of Otago, Dunedin, NZ). Mice were maintained in either open top or individually ventilated cages with shredded paper nesting in environmentally controlled rooms (temperature 22°C ± 1°C, 12 h light/dark cycle) with ad libitum access to food and water.

#### 2.2.1 Mouse strains

C57BL/6J mice were sourced from the University of Otago colony in which mouse stock is routinely replenished from Jackson Laboratory (IMSR Cat# JAX:000664, RRID:IMSR JAX:000664, The Jackson Laboratory, Bar Harbor, Maine, USA). *Igf2* ^*fl/fl*^ mice^22^ (loxP sites flank exons 4–6 of the *Igf2* gene) were kindly provided by Drs. Miguel Constância and Ionel Sandovici (University of Cambridge, UK). *Prlr‐*internal ribosome entry site (IRES)‐Cre mice were generated by our lab in collaboration with Ulrich Boehm, Saarland University, and sourced from our University of Otago colony. Details regarding the generation of this strain are available in Kokay et al., 2018.^23^ All transgenic mouse strains used in this study were maintained on a C57BL/6J background.

### 2.3 Cerebrospinal fluid sampling across reproductive states

#### 2.3.1 CSF collection

Adult female C57BL/6J mice (*N* = 49), aged 8–10 weeks at the start of the experiment were individually housed in open‐top cages. Mice were split into five groups: virgin (diestrus, *n* = 16), early (Day 7, *n* = 7) and late (Day 18, *n* = 8) pregnancy, and lactating (Days 9–12, *n* = 18). For timed mating, confirmation of the presence of a vaginal plug was designated pregnancy Day 1, and the day of birth was recorded as lactation Day 1. For cycling (virgin) mice, vaginal cytology was monitored for two consecutive estrous cycles, and collection of CSF was performed on diestrus of the third consecutive cycle. CSF (10–20 μL, per mouse) was collected between 1000 and 1600 h, based on the procedure described by Liu and Duff (2008) with modifications.^24^ In brief, mice were anaesthetized (i.p.) using a mixture of ketamine (75 mg/kg) and domitor (1 mg/kg) and placed in a stereotaxic frame. A sagittal incision was made inferior to the occiput, and blunt dissection was employed to separate the underlying subcutaneous and muscle tissue (*m biventer cervicis* and *m rectus capitis dorsalis major*) revealing the cisterna magna. A pulled glass microcapillary tube was inserted into the cisterna magna to collect CSF. The microcapillary tube was then carefully removed, and CSF was emptied into a sterile microcentrifuge tube, then immediately frozen on dry ice and stored at −80°C.

#### 2.3.2 IGF2 ELISA

Levels of IGF2 were measured in CSF samples collected from adult female C57BL/6J mice in 2.3.1, using a sensitive IGF2 ELISA kit (SEA051Mu, Cloud‐Clone Corp, TX, USA) in accordance with the manufacturer's instructions. CSF was diluted 1:25 in 0.01 M phosphate buffered saline (PBS). Then 100 μL of each diluted sample was loaded into assay plates in duplicate. The assay detection range is 15.6–1000 pg/mL and the lower limit of sensitivity is <6.0 pg/mL. Intra‐assay and inter‐assay coefficients were <10%.

### 2.4 IGF2 immunohistochemistry and *Igf2/Prlr* RNAscope assay

#### 2.4.1 Tissue collection and sectioning

Adult female C57BL/6J mice in diestrus were deeply anaesthetized with pentabarbitone (i.p., 100 mg/kg) prior to transcardial perfusion with 2% (for RNAscope, *n* = 6) or 4% w/v (for immunohistochemistry, *n* = 6) formaldehyde in 0.1 M phosphate buffer (PB). Brains were collected, post‐fixed for 1 h in 2% or 4% w/v formaldehyde respectively, immersed in 30% w/v sucrose for 48 h, and frozen at −80°C. Three serial sets of sections (14 μm thickness) were float‐mounted in milli Q water onto Superfrost plus slides and stored at −80°C.

#### 2.4.2 Immunohistochemistry

Sections were thawed and taken through three washes in 0.01 M Tris‐buffered saline (TBS), then endogenous peroxidases were blocked with 3% hydrogen peroxide (H_2_O_2_). Blocking solution (0.05 M TBS, 0.3% Triton X‐100, 0.25% Bovine Serum Albumin (BSA), 2% normal goat serum (NGS)) was applied to sections for 1 h to prevent nonspecific binding of antibodies. Then, sections were incubated for 48 h in blocking solution containing rabbit polyclonal anti‐IGF2 (ab63984, Abcam, Cambridge, UK) diluted 1:200. Blocking solution was applied to negative control sections. Sections were washed and then incubated with biotinylated goat anti‐rabbit (BA‐1000, Vector Laboratories, Burlingame, CA, USA) for 2.5 h, followed by Avidin/Biotin complex (A/B Vectastain Elite, Vector Laboratories) for 1.5 h. Sections were washed in 0.01 M TBS, rinsed in 0.1 M sodium acetate, and reacted with glucose oxidase‐catalyzed nickel‐enhanced 3,3′‐diaminobenzidine (DAB) to reveal positive IGF2 immunoreactivity. Sections were dehydrated through an ethanol series to xylene and coverslipped with dibutylphthalate polystyrene xylene (DPX). Images to visualize expression patterns were taken with an AX70 Provis light microscope (Olympus, Tokyo, Japan) and attached Spot RT digital camera (Spot imaging, Sterling Heights, MI).

#### 2.4.3 *Igf2* and *Prlr* RNAscope

*Igf2* and *Prlr* in situ hybridization was performed using an RNAscope 2.5 high definition Assay kit‐RED (Advanced Cell Diagnostics, Hayward, CA) as previously published.^25^ In brief, sections were thawed at 55°C for 5 min, post‐fixed in 2% w/v PFA, and washed in 0.01 M PBS. Endogenous peroxidases were blocked with RNAscope H_2_O_2_ solution prior to immersion in 100% ethanol. Tissue was permeabilized with RNAscope protease plus solution for 30 min at 40°C. Sections were hybridized for 2 h at 40°C with either an *Igf2* or *Prlr* probe. The *Igf2* probe (Ca# 437671) targets the region spanning 432–1625 bps in transcript NM_010514.3. The *Prlr* probe (Ca# 430791) is designed to transcript NM_011169.5 with target region 636–1640 bps. The runs also included sections hybridized with a negative DapB control probe (Ca# 310043), designed to transcript EF191515 with target region 414–862 bps. Following hybridization, sections were taken through a series of amplification steps using Amp solutions 1–6 with the manufacturer's designated incubation times and wash steps. After the last amplification incubation, sections were washed and positive hybridization was detected by incubating sections with Fast‐RED B:Fast‐RED A (1:60) detection reagents for 10 min at RT. Sections were counterstained with hematoxylin and coverslipped with Ecomount (Biocare Medical, Pacheco, CA). Images were taken using the same microscope as for IGF2 immunohistochemistry (2.4.2). Positive hybridization for *Igf2* and *Prlr* was determined based on scoring guidelines included in the manufacturer's protocol (Advanced Cell Diagnostics).

### 2.5 Behavioral testing

#### 2.5.1 *Igf2* mouse models

Two conditional *Igf2* knockout mouse models were used in this study to assess the effects of *Igf2* removal from CP. In the first model, *Igf2* ^*fl/fl*^ mice were crossed with *Prlr*‐IRES‐Cre to generate *Igf2 Prlr*‐IRES‐Cre mice (Cre+ *n* = 11 and Cre− *n* = 10) in which *Igf2* is conditionally removed for all *Prlr‐*containing cells. Homozygous deletion of the two *Igf2* floxed alleles was achieved through two rounds of breeding, with a third round added to limit animal wastage by providing all female offspring with the preferred genotype. The first cross involved mating a homozygous *Igf2* ^*fl/fl*^ female mouse with a homozygous *Prlr IRES‐*Cre^+/+^ male mouse to generate offspring with deletion of a single *Igf2* allele in *Prlr*‐containing cells (*Igf2* ^*fl/−*^ *Prlr IRES*‐Cre^+/−^). Double heterozygous offspring were then mated with a homozygous *Igf2* floxed mouse (*Igf2* ^*fl/−*^ *Prlr IRES*‐Cre^+/−^ (male mouse) × *Igf2* ^*fl/fl*^ (female mouse)) to generate approximately 25% of offspring with homozygous deletion of *Igf2* in *Prlr*‐containing cells (*Igf2* ^fl/fl^ *Prlr IRES*‐Cre^+/−^). The third round of breeding crossed a homozygous *Igf2* floxed mouse heterozygous for Cre recombinase with a homozygous *Igf2* floxed mouse (*Igf2* ^*fl/fl*^ *Prlr IRES‐*Cre^+/−^ (male mouse) × *Igf2* ^*fl/fl*^ (female mouse)) to generate female offspring with the genotypes *Igf2* ^*fl/fl*^ *Prlr‐IRES* Cre^+/−^ and *Igf2* ^*fl/fl*^ *Prlr‐IRES* Cre^−/−^ to be used as experimental and control animals, respectively, in this study.

The second mouse model employed adeno‐associated virus (AAV) delivery of Cre recombinase into the lateral ventricles to conditionally remove *Igf2* specifically from the CP. Adult virgin *Igf2* ^fl/fl^ mice (6 weeks old, *N* = 20) were anaesthetized with isofluorane and placed in a stereotaxic frame. Bilateral (1 μL) intracerebroventricular injections of pAAV.CMV.HI.eGFP‐Cre WPRE SV40 (AAV5, 1 × 10^13^ Gc/mL, Addgene Watertown, MA, USA, *n* = 10) were administered. The control group received AAV/DJ.CMV.mCherry (3.7 × 10^13^ Gc/ml, Vector Biolabs, Malvern, PA, USA, *n* = 10). All injections were carried out at 100 nL/min and needles were left in place for 3 min prior to and 10 min after injection. Coordinates for ICV injection were bregma ±0.07 mm (lateral) and −0.35 mm (dorsal). Mice were left to recover for 4 weeks post‐surgery, prior to any further manipulations.

#### 2.5.2 Animal husbandry and testing parameters

All mice were single housed in IVC cages under a reverse light cycle (12 h dark/light cycle) with sodium light. Mice were weighed daily across the virgin, pregnant, and lactating states, and litters were weighed through lactation. All tests were performed during the dark (active) phase, and researchers were blinded to genotype. Mice were tested for alterations in olfaction in their virgin state using a buried food test^26^ to provide an indication of the presence or absence of disruption to olfactory neurogenesis in our *Igf2* conditional knockout mouse models. All mice previously tested in the buried food test were mated with a C57BL/6J male mouse. Successful pregnancy was confirmed by the presence of a vaginal plug (recorded as pregnancy Day 1) and the male was removed. The day of parturition was designated as lactation Day 1. On Day 2 of lactation, the number of live pups in each litter was recorded, and litter size was limited to *n* = 6. At the same time, observations relating to maternal care were recorded. These included litter deaths, the presence/absence of milk spots in the abdomen of pups as a measure of successful milk production, and whether a well‐formed nest was present. Two subsequent behavioral tests, the elevated plus maze and light/dark transition test, were performed during early lactation to identify any changes in anxiety‐like behavior exhibited in the different *Igf2* conditional knockout mouse models post‐partum. The test apparatus was sanitized after each mouse with 70% ethanol to remove fecal matter and any olfactory clues.

#### 2.5.3 Buried food test (BFT)

Virgin *Igf2 Prlr*‐IRES‐Cre and *Igf2* ^fl/fl^ AAV cre mice were habituated to handling for 2 weeks before being subjected to a buried food test in accordance with the protocol described by Yang and Crawley (2009).^26^ Vaginal cytology was monitored for 2 cycles, and testing was performed on the day of diestrus in the third consecutive cycle between 1000 and 1400 h. Odor familiarization was established, and palatability was checked by placing a fruit loop (a high‐sugar breakfast cereal) in the cage on the days of proestrus and estrus in the third consecutive cycle. On the day prior to testing, all food was removed, and mice were fasted for 18 h. On test day, mice were habituated to the testing room for 30 min, then placed in a novel cage for 5 min of acclimation. Mice were then moved to a temporary clean cage, while a fruit loop was buried in the novel cage they had just vacated. Mice were reintroduced to the novel cage with the fruit loop now buried, and behavior was recorded for 15 min under sodium light (127 Lux). At the end of the test, mice were rehoused in their home cage. Latency to find the fruit loop, defined as uncovering the fruit loop and holding it in both forelimbs, and time spent eating were recorded.

#### 2.5.4 Elevated plus maze (EPM)

In the EPM, mice prefer the dark enclosed arms, but less anxious mice will spend more time exploring the open lighter arms.^27^ It has been well characterized that post‐partum mice normally show markedly reduced anxiety‐like behavior on this test, as compared to nonpregnant mice.^28^, ^29^ Mice were habituated to the testing room for ≥30 min under sodium light. At the start of the test, each mouse was placed in the center of the maze facing an open arm, and white light was turned on to 305 Lux. Behavior was recorded for 5 min under white light, and analysis of recordings was performed using TopScan (CleverSys Inc). Behavioral parameters measured included the number of bouts on open arms, total time spent on open arms, distance traveled on open arms, and the total distance traveled in the EPM. An entry onto an open or closed arm was defined in our analysis as 75% of the mouse's body (approximately three of the four paws) being on the appropriate arm.

#### 2.5.5 Light/dark transition test

The light/dark transition test uses a box with a dark and light chamber.^30^ Since mice have a natural aversion to bright light, mice are put in the dark chamber, and the time taken to initially enter the light chamber acts as a testable measure to assess anxiety levels.^30^ This test was performed on Day 5 of lactation on all *Igf2* conditional knockout mice. Mice were habituated to the testing room for ≥30 min under sodium light. At the start of the test, each mouse was placed in the dark chamber, and white light (305 Lux) turned on. Behavior was recorded for 5 min, and behavioral components, such as total time spent in the light chamber, number of entries into the light chamber, and latency to enter the light chamber, were analyzed. An entry was recorded when all four of the mouse's paws were in the same chamber.

### 2.6 Real time PCR

To evaluate the extent of *Igf2* expression in CP tissue, *Igf2* conditional knockout mice were decapitated at the end of the study and brains removed. RNA was extracted from CP dissected from the lateral ventricle in each brain using our previously described method.^6^ First strand cDNA synthesis was carried out using Quantabio qscript XLT cDNA SuperMix (95161–100, dnature diagnostics and research Ltd., Gisborne, NZ) in accordance with the manufacturer's protocol. DNase‐treated total RNA (50 ng) from each sample was used as a template. qRT‐PCR was performed with PowerUp™ SYBR™ Green Master Mix (A25741, Thermo Fisher Scientific, Waltham, MA, USA) and custom‐made primers for *Igf2*, *Prlr* (to detect all forms), and *Prlr* (to detect long form only) previously described in reference 6 were designed using Primer‐Basic Local Alignment Search Tool (BLAST; http://blast.ncbi.nlm.nih.gov/Blast.cgi) (Table 1). Samples (10 ng cDNA) were run as duplicate reactions (10 μL) and no template and RNA template controls were included. qRT‐PCR plates (96 well) were run in an Applied Biosystems ViiA 7 Real‐time PCR system (Thermo Fisher Scientific) using the thermocycling protocol 50°C for 2 min, 95°C for 2 min, then 40 cycles of 95°C for 15 sec and 60°C for 1 min. A melt curve was included post‐amplification. *Gapdh* and *Actb* (primer sequences, Table 1), selected based on their gene stability scores using refinder (https://blooge.cn/RefFinder/) were used as reference genes.

**TABLE 1:** Quantitative PCR primer sets used for validation of *Igf2* knockout mouse models.

Gene symbol	Sequence (5′–3′)^a^	Amplicon	Accession number/nucleotide location	Efficiencies	Final concentration
*Prlr* (all forms)	F‐ATGTCGTTCCCCTGACAAGGA R‐GAATTGGGGCCACTGGTTTTG	150 bp	NM_011169 F 838–858 R 987–967	1.04	F 500 nM R 800 nM
*Prlr* (long form only)	F‐GCATGATGACCTGCATCTTTCC R‐CCAAGGCACTCAGCAGTTCTT	104 bp	NM_011169 F 1518–1539 R 1622–1602	1.03	F 800 nM R 800 nM
*Igf2*	F‐ACACGCTTCAGTTTGTCTGTTC R‐GGGGGTGGCACAGTATGTC	146 bp	NM_010514 F 563–584 R 708–690	1.03	F 500 nM R 500 nM
*Actb*	F‐AGGCCAACCGTGAAAAGATG R‐GCCTGGATGGCTACGTACATG	76 bp	NM_007393 F 447–466 R 522–502	1.01	F 800 nM R 800 nM
*Gapdh*	F‐TTCAACAGCAACTCCCACTCTT RGCCGTATTCATTGTCATACCAG	102 bp	NM_008084 F 1090–1111 R 1191–1170	0.99	F 800 nM R 800 nM

^a^ All primer sequences previously described in reference 6.

### 2.7 SVZ mitogenesis

Treatment with Bromodeoxyuridine (BrdU), a thymidine analogue, has been used previously to show a peak in cell proliferation in the SVZ early in lactation.^10^ Hence, in our study, cohorts of C57BL/6J (*n* = 23) and *Igf2 Prlr*‐IRES‐Cre mice (*n* = 29) were injected twice (2 h apart) with BrdU (i.p., 12 mg/100 g bodyweight), either on diestrus or during lactation (Days 7–9) to measure cell proliferation. Two hours following the final injection, mice were transcardially perfused with 4% w/v PFA in 0.1 M PB. Brains were collected as described in 2.4.1.

#### 2.7.1 BrdU immunohistochemistry

Four sets of serial cryosections (30 μm thick) were cut and stored in cryoprotectant at −20°C until processing. Free‐floating sections were immersed in incubation solution (0.05 M TBS, 0.3% Triton X‐100, 0.25% BSA) for 10 min, prior to antigen retrieval in a 2 M hydrochloric acid solution at 37°C for 30 min. Sections were washed repeatedly in incubation buffer, then 0.01 M TBS. Endogenous peroxidases were blocked using 30% H_2_O_2_ solution, then immersed in incubation solution with 5% NGS as a secondary blocking step. Sections were incubated with endogenous mouse IgG (1:100 in 0.01 M TBS, ca# 115‐007‐003, Jackson ImmunoResearch, PA, USA) overnight at 4°C, prior to 48 h incubation with mouse anti‐BrdU (Ca# M0744, anti BrdU Clone Bu20a, Dako, Denmark) 1:1000 in incubation solution with 2% NGS. Negative control sections were incubated with incubation solution containing 2% NGS only. The primary antibody was removed and replaced for 1.5 h at RT with biotinylated goat anti‐mouse IgG (Ca# BA‐9200, Vector Laboratories) 1:500 in incubation solution on all sections. Sections were incubated with Avidin/Biotin Vectastain Elite solution (Ca #PK‐6100, Vector Laboratories) for 1.5 h at RT, then reacted with glucose oxidase‐catalyzed nickel‐enhanced 3,3′‐DAB to reveal BrdU immunopositive cells. Sections were mounted onto gelatin‐coated slides and coverslipped. For SVZ mitogenesis analysis, systematic quantification of BrdU+ cell counts was performed on 14 sections (120 μm apart, between bregma ~1.18 mm to ~−2.54 mm with random start) through the SVZ using an Olympus AX70 microscope under 20× objective. Cells were classified as positive if the entire nuclear profile of the labeled nucleus was evident in the field of view. Positive cell counts on the dorsal, septal, and lateral sides of both ventricles were recorded, and researchers were blinded to genotype and treatment groups.

### 2.8 Statistical analysis

All data, except qPCR results, are reported as mean ± SEM, and statistical analyses were performed using GraphPad Prism 9 (v9.5.1; GraphPad Software, LLC). In all cases, *p* < .05 was considered statistically significant, and data were checked for normality using D'Agostino–Pearson omnibus, Anderson–Darling, Shapiro–Wilk and Kolmogorov–Smirnov normality tests as appropriate, and an *F*‐test for equal variance. All result data were tested for outliers using ROUT (*Q* = 1%) and if outliers were identified they were removed from downstream analysis. For IGF2 levels measured in CSF by ELISA, one‐way ANOVA with a Tukey post hoc test was used to statistically compare IGF2 levels across different reproductive states. Body weight data from *Igf2* conditional knockout mice through different reproductive states were analyzed by repeated measures two‐way ANOVA with Tukey multiple comparisons test to identify any statistically significant changes between genotypes over time. For behavioral testing, BFT, EPM and light/dark transition test, unpaired *t*‐tests (Welch's *t*‐test and Mann–Whitney tests when appropriate) were used to compare different behavioral parameters between controls and Cre‐expressing groups. Comparisons between BrdU+ cell counts through the SVZ between diestrus and lactating groups were analyzed using unpaired *t*‐tests and one‐way ANOVAs with Tukey post hoc test where appropriate. Raw qPCR data were analyzed using ViiA 7 Software, and resultant CT values were analyzed using the 2^−ΔΔCt^ method as previously described in Phillipps et al.^31^ Data are presented as changes in relative gene expression with respect to the control groups, either *Igf2 Prlr*‐IRES‐Cre− or *Igf2* ^fl/fl^ mCherry ± SEM.

## 3 RESULTS

### 3.1 IGF2 mRNA and protein expression in SVZ neurogenic niche

In the SVZ neurogenic niche IGF2 is abundant in CSF and *Prlr* and *Igf2* are highly expressed in epithelial cells of the CP (Figure 1B–E). *Igf2* expression is also evident in brain endothelial cells (Figure 1B, B1, B2). IGF2 levels in CSF remained relatively stable in the transition from the virgin to pregnant state and during pregnancy, but were significantly elevated in lactation (one‐way ANOVA with Tukey post‐hoc test, *n* = 18, *F*(3, 45) = 9.607, *p* < .001) compared to both pregnancy (Day 7 preg, *n* = 7; *p* = .0015; Day 18 preg, *n* = 8; *p* = .0003) and virgin (diestrus, *n* = 16; *p* = .0098) states (Figure 1E). Additionally, IGF2 levels in CSF were more variable in lactation (*F*(3, 45) = 6.437; *p* = .0010, Figure 1E).

### 3.2 Characterization of conditional *Igf2* knockout mice

**FIGURE 2:**
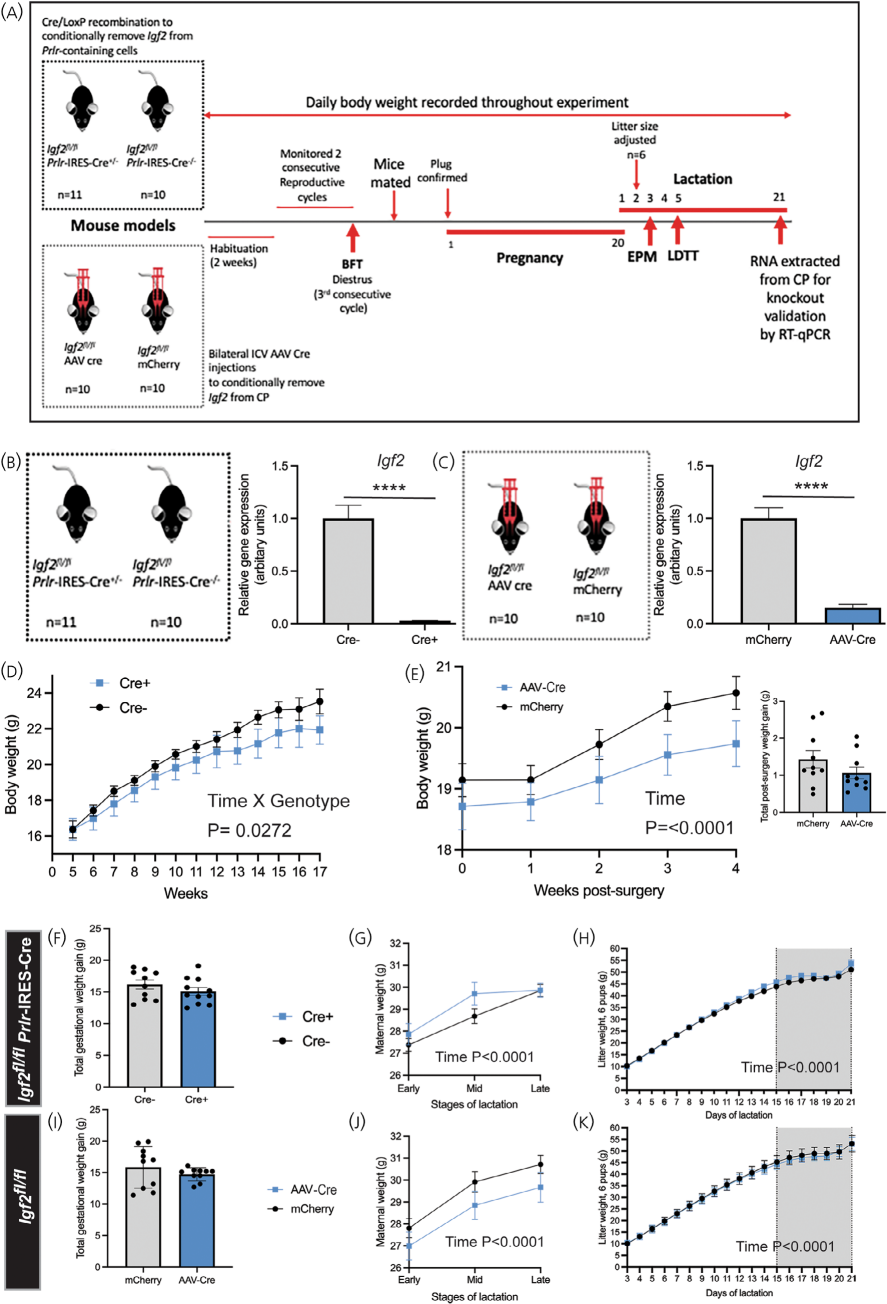
Characterization of conditional *Igf2* knockout mouse models. (A) Experimental timeline showing all testing and procedures carried out using the two mouse models, *Igf2* ^*fl/fl*^ *Prlr*‐IRES‐Cre (Cre+ *n* = 11, Cre− *n* = 10) and *Igf2* ^*fl/fl*^ (AAV‐Cre/mCherry, *n* = 10 each). (B and C) qPCR validation of *Igf2* expression in CP collected from *Igf2* ^*fl/fl*^ *Prlr*‐IRES‐Cre (Cre+ and Cre−, B) and *Igf2* ^*fl/fl*^ (AAV‐Cre/mCherry, C) mice. *p* < .0001, unpaired two‐tailed *t* test, data presented as changes in relative gene expression with respect to control groups. (D) Mean weekly body weights taken from *Igf2* ^*fl/fl*^ *Prlr*‐IRES‐Cre (Cre+ and Cre−) from 5 to 17 weeks of age. Note the reduced body weight observed in *Igf2* ^*fl/fl*^ *Prlr*‐IRES‐Cre^+/−^ mice (Time × Genotype *p* = .0272; two‐way ANOVA with repeated measures). (E) Mean weekly body weights taken from virgin *Igf2* ^*fl/fl*^ (AAV‐Cre/mCherry) over 4 week period post‐surgery (Time *p* < .0001, two‐way ANOVA with repeated measures). Bar graph shows total post‐surgery weight gain across the 4 weeks in the same mice (*p* = .2134, unpaired *t* test). (F and I) Total weight gain through pregnancy (Days 1–18) was similar between genotypes, *Igf2* ^*fl/fl*^ *Prlr*‐IRES‐Cre (Cre+ and Cre−, F, *p* = .2609, unpaired *t* test) mice and *Igf2* ^*fl/fl*^ (AAV‐Cre/mCherry, I, *p* = .3315, unpaired *t* test). (G and J) Mean maternal body weights obtained for early (Lactation days (LD) 1–7), mid (LD Days 8–14) and late (LD 15–21) lactation for both *Igf2* ^*fl/fl*^ *Prlr*‐IRES‐Cre (Cre+ and Cre−, G) and *Igf2* ^*fl/fl*^ (AAV‐Cre/mCherry, J) mice (Time *p* < .0001, two‐way ANOVA with repeated measures). Litter weights obtained from *Igf2* ^*fl/fl*^ *Prlr*‐IRES‐Cre mothers (H) and *Igf2* ^*fl/fl*^ (AAV‐Cre/mCherry) mothers (K), (Time *p* < .0001, two‐way ANOVA with repeated measures). Data in D–K presented as mean ± SEM.

To analyze the role of *Igf2* in the CP, we developed two conditional *Igf2* knockout mouse models (Figure 2A). For one model, we crossed *Igf2* ^*fl/fl*^ mice^32^ with a mouse line targeting Cre recombinase expression specifically to *Prlr*‐containing cells, *Prlr‐*IRES‐Cre.^23^, ^33^ We used a two‐generation breeding strategy (outlined in 2.5.1) to generate a mouse line in which *Igf2* is conditionally removed from all *Prlr*‐containing cells (Figures 2B and S1). For our second model, to specifically remove *Igf2* solely from the CP, we stereotaxically injected a recombinant AAV‐Cre or AAV encoding Cre‐dependent mCherry (control) into both lateral ventricles to specifically target CP tissue in adult mice (Figure 2C). The advantage of this model is that both *Igf2* floxed alleles were deleted simultaneously, eliminating the need for a two‐generation cross, and there was no potential confound of loss of *Igf2* in other *Prlr*‐expressing tissues. Both models showed substantial knockdown in *Igf2* levels in CP tissue (unpaired two‐tailed *t* test, *Igf2* ^*fl/fl*^ *Prlr*‐IRES‐Cre^+/−^ mice, *t*(19) = 8.307, *p* < .0001; *Igf2* ^*fl/fl*^ AAV‐Cre/mCherry mice *t*(18) = 9.390, *p* < .0001; Figure 2B,C). Interestingly, *Igf2* ^*fl/fl*^ *Prlr*‐IRES‐Cre^+/−^ mice also showed a reduction in body weight gain over time as compared to controls in the virgin state (two‐way ANOVA with repeated measures: Time × Genotype *F*(12,180) = 1.993, *p* = .0272; Figure 2D). Genotype‐specific changes in body weight gain over time were not observed in virgin *Igf2* ^*fl/fl*^ AAV‐Cre/mCherry mice, but rather an expected increase over time in both genotypes was recorded (two‐way ANOVA with repeated measures: Time *F*(92.046, 36.82) = 44.57, *p* = <.0001; Figure 2E). No changes in overall weight gain between knockouts and controls were observed in either model during pregnancy or lactation (Figure 2F,G,I,J). In addition, milk spots were observed in all live pups, and litter weights were also consistent between knockouts and controls, and as anticipated, an increase over time was observed (two‐way ANOVA with repeated measures, *Igf2* ^*fl/fl*^ *Prlr*‐IRES‐Cre^+/−^ mice: Time *F*(1.390, 26.41) = 1812, *p* < .001; *Igf2* ^*fl/fl*^ AAV‐Cre/mCherry mice: Time *F*(1.260, 28.99) = 4820, *p* < .0001; Figure 2H,K). Finally, well‐formed nests were observed for all mice, and two litter deaths were recorded, one from a mouse in which *Igf2* has been removed from all *Prlr*‐containing cells (*Igf2* ^*fl/fl*^ *Prlr*‐IRES‐Cre^+/−^) the other from a control mouse (*Igf2* ^*fl/fl*^ *Prlr*‐IRES‐Cre^−/−^); consequently, these mice were excluded from the study.

### 3.3 Behavioral testing in virgin and lactating states

#### 3.3.1 Olfaction testing

**FIGURE 3:**
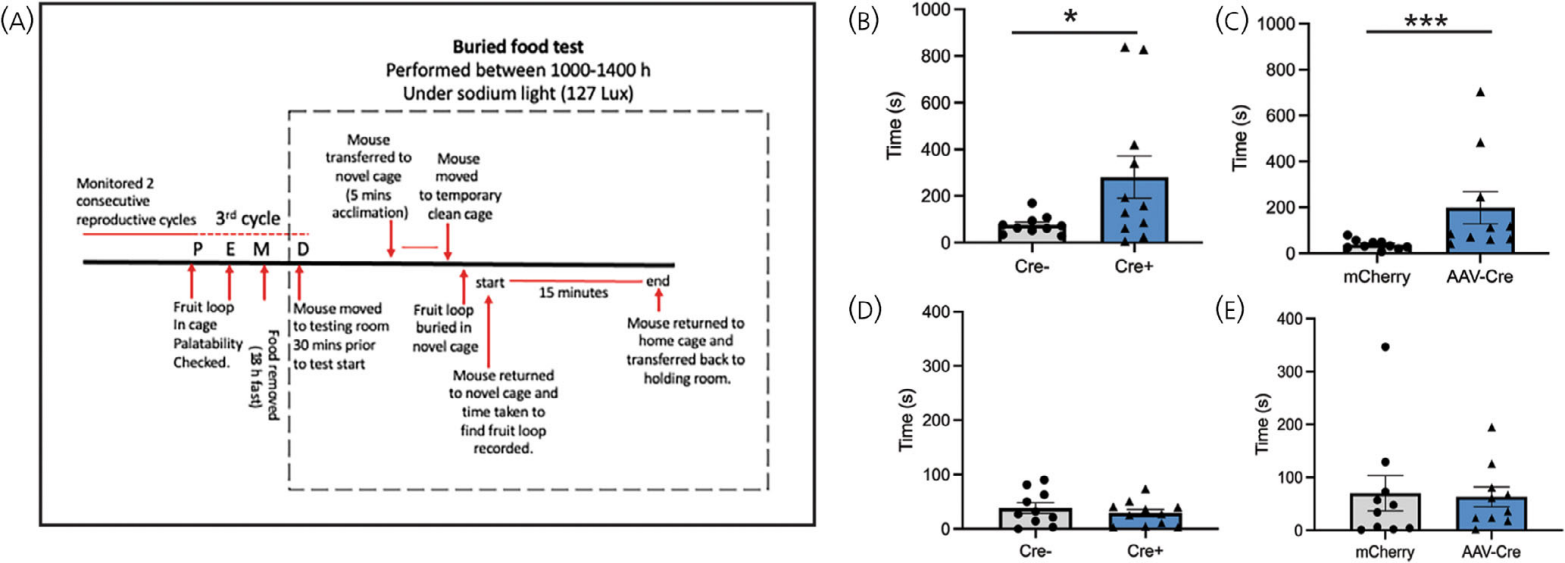
Olfaction deficits in virgin *Igf2* knockout mouse models. (A) Experimental timeline showing steps carried out in the buried food test when assessing the two mouse models, *Igf2* ^*fl/fl*^ *Prlr*‐IRES‐Cre (Cre+ *n* = 11, Cre− *n* = 10) and *Igf2* ^*fl/fl*^ (AAV‐Cre/mCherry, *n* = 10 each). Graphs (B/C) show *Igf2* ^*fl/fl*^ *Prlr*‐IRES‐Cre^+/−^ (B) and *Igf2* ^*fl/fl*^ AAV‐Cre (C) mice take significantly longer to find a buried fruit loop as compared to controls. (*F* test [F, DFn, Dfd 116.9, 9, 9, *p* < .0001], Welch's *t*‐test *p* = .0479, (Figure 3B) and Mann–Whitney test *p* = .0005 (Figure 3C)). Graphs (D/E) show no differences across genotypes in the time spent eating the fruit loop once revealed, *Igf2* ^*fl/fl*^ *Prlr*‐IRES‐Cre^+/−^ (D) and *Igf2* ^*fl/fl*^ (AAV‐Cre/mCherry, E). Unpaired *t*‐tests were used to assess differences between knockout and control groups and data are presented as mean ± SEM.

Since IGF2 is a critical regulator of NSC pool maintenance in the SVZ and neuroblasts arising in the SVZ migrate rostrally to integrate into circuitry within the olfactory bulb, we sought to determine whether virgin *Igf2* knockout mice showed any deficits in their ability to smell volatile odors. Using the buried food test (Figure 3A),^26^ we observed both *Igf2* ^*fl/fl*^ *Prlr*‐IRES‐Cre+ and *Igf2* ^*fl/fl*^ AAV‐Cre mice took longer to uncover a buried fruit loop following an 18 h fasting period as compared to controls (Welch's *t* test *Igf2* ^*fl/fl*^ *Prlr*‐IRES‐Cre^+/−^ mice: *t*(10.4) = 2.242, *p* = .0478 (Figure 3B)); *Igf2* ^*fl/fl*^ AAV‐Cre/mCherry mice (Mann–Whitney *U* test, *U* = 7, *p* = .0005 (Figure 3C)). Once uncovered, the time spent eating the fruit loop was comparable across genotypes (*Igf2* ^*fl/fl*^ *Prlr*‐IRES‐Cre^+/−^ mice: Welch's *t*‐test, *t*(16.11) = 0.7777, *p* = .4480 (Figure 3D)) and *Igf2* ^*fl/fl*^ AAV‐Cre/mCherry mice: Mann–Whitney *U* test, *U* = 43, *p* = .6305 (Figure 3E).

#### 3.3.2 Anxiety behavioral testing

**FIGURE 4:**
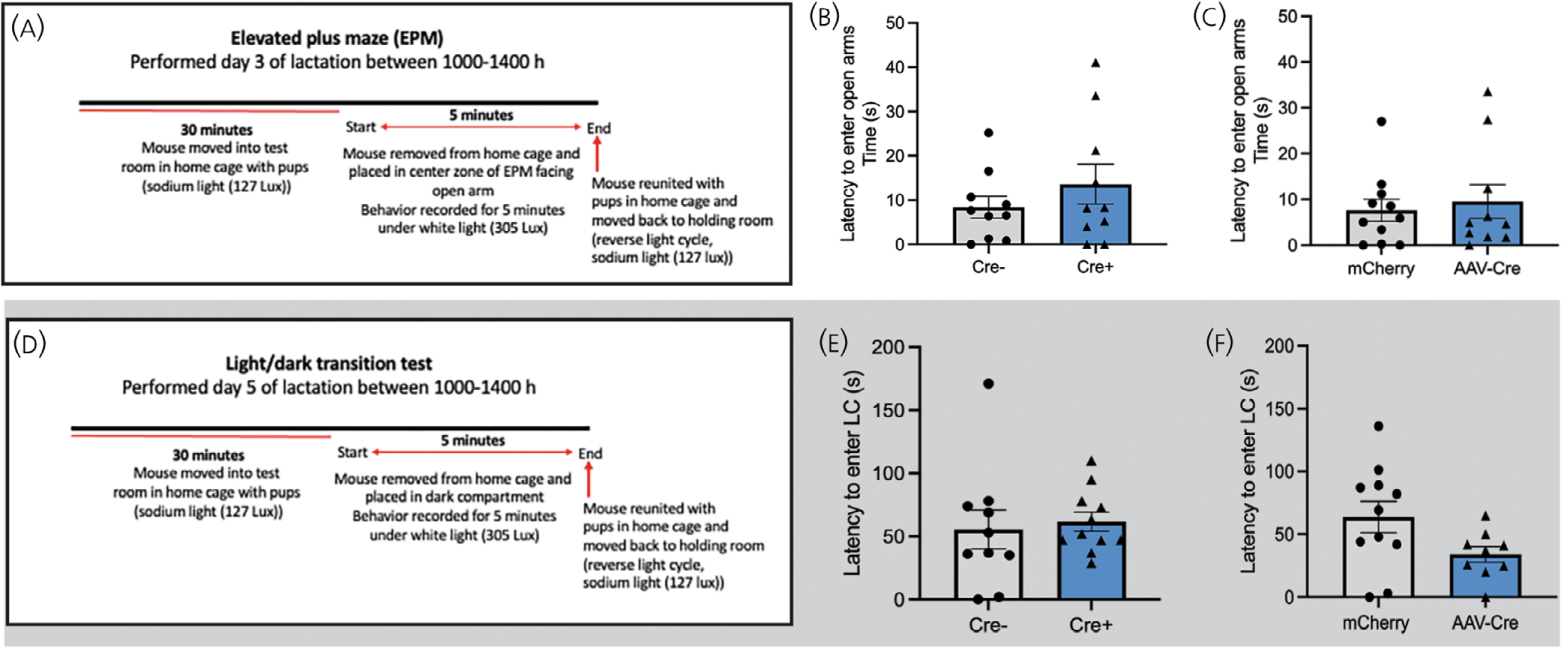
Maintenance of post‐partum mood resilience. Testing timelines for EPM (A) and Light/dark transition (D) tests. No significant differences were found between genotypes of *Igf2* ^*fl/fl*^ *Prlr*‐IRES‐Cre (Cre+ *n* = 11, Cre− *n* = 10) or *Igf2* ^*fl/fl*^ (AAV‐Cre/mCherry, *n* = 10 each) across all parameters measured in either the EPM (B, C) or light/dark transition (E, F) tests. Graphs (B/C) show the latency taken for *Igf2* ^*fl/fl*^ *Prlr*‐IRES‐Cre (B) and *Igf2* ^*fl/fl*^ (AAV‐Cre/mCherry) (C) mice to enter open arms in the EPM. Graphs (E/F) show the latency to enter the light chamber for *Igf2* ^*fl/fl*^ *Prlr*‐IRES‐Cre (E) and *Igf2* ^*fl/fl*^ (AAV‐Cre/mCherry) (F) mice. Unpaired *t*‐tests were used to assess differences between knockout and control groups and data are presented as mean ± SEM. LC, light chamber.

Alterations in prolactin levels early in pregnancy have been attributed to increased anxiety‐like behavior post‐partum. We have previously shown prolactin‐induced *Igf2* expression in the CP.^6^ Hence, we performed elevated plus maze (EPM) (Figure 4A) and light/dark transition tests (Figure 4D) to assess the effect of IGF2 loss exclusively in both *Prlr*‐containing cells or CP tissue on post‐partum anxiety levels. For the EPM, mice were placed in the center zone of the maze on Day 3 of lactation. Entry to the open arms, time spent on the open arms, and distance traveled in the maze were recorded and evaluated over a 5 min period. No differences in measured parameters were observed between each of the genotypes and their controls for both mouse models either with *Igf2* removal solely in *Prlr*‐containing cells (Latency to enter open arms: Welch's *t*‐test, *t*(13.96) = 1.014, *p* = .3277) (Figure 4B); Number of bouts on open arms: unpaired *t*‐test, *t*(19) = 2.077, *p* = .0516 (Figure S2A); Time spent on open arms: unpaired *t*‐test *t*(19) = 1.586, *p* = .1292, (Figure S2C); Distance traveled on open arms: Welch's *t*‐test *t*(13.41) = 1.846, *p* = .0870 (Figure S2E); Total distance traveled: unpaired *t*‐test, *t*(19) = 1.488, *p* = .1531 (Figure S2G) or exclusive *Igf2* knockout in the CP (Latency to enter open arms: *p* = .6681, *t* = .4370, DF = 15.64, (Figure 4C); Number of bouts on open arms: Welch's *t*‐test, *t*(19) = 0.2476, *p* = .8071 (Figure S2B); Time spent on open arms: unpaired *t*‐test, *t*(19) = 0.4168, *p* = .6815 (Figure S2D); Distance traveled on open arms: Welch's *t*‐test, *t*(16.7) = 0.3804, *p* = .7984 (Figure S2F); Total distance traveled: unpaired *t*‐test, *t*(19) = 1.817, *p* = .0851 (Figure S2H)). The same mice tested in the EPM on Day 3 of lactation were then further assessed for anxiety‐like behavior on Day 5 of lactation using the light/dark transition test. Mice were placed in the dark chamber, and the time taken to enter the light chamber, the number of entries to the light chamber, and the total time spent in the light chamber were recorded over a 5 min period. Overall, no differences in anxiety levels were observed between the two *Igf2* knockout mouse models and their controls (*Igf2* ^*fl/fl*^ *Prlr*‐IRES‐Cre^+/−^ mice: Latency to enter light chamber: Welch's *t*‐test, *t*(13.14) = 0.3575, *p* = .7264 (Figure 4E); Number of entries into light chamber: unpaired *t*‐test, *t*(19) = 0.1713, *p* = .8658 (Figure S2I); Total time spent in light chamber: Welch's *t*‐test, *t*(17.77) = 0.3509, *p* = .7298 (Figure S2K); *Igf2* ^*fl/fl*^ AAV‐Cre/mCherry mice: Latency to enter light chamber: Welch's *t*‐test, *t*(14.56) = 2.136, *p* = .0501 (Figure 4F); Number of entries into light chamber: Welch's *t*‐test, *t*(15.68) = 1.383, *p* = .1860 (Figure S2J); Total time spent in light chamber: Welch's *t*‐test, *t*(11.21) = 1.928, *p* = .0795 (Figure S2L)).

### 3.4 SVZ proliferation

**FIGURE 5:**
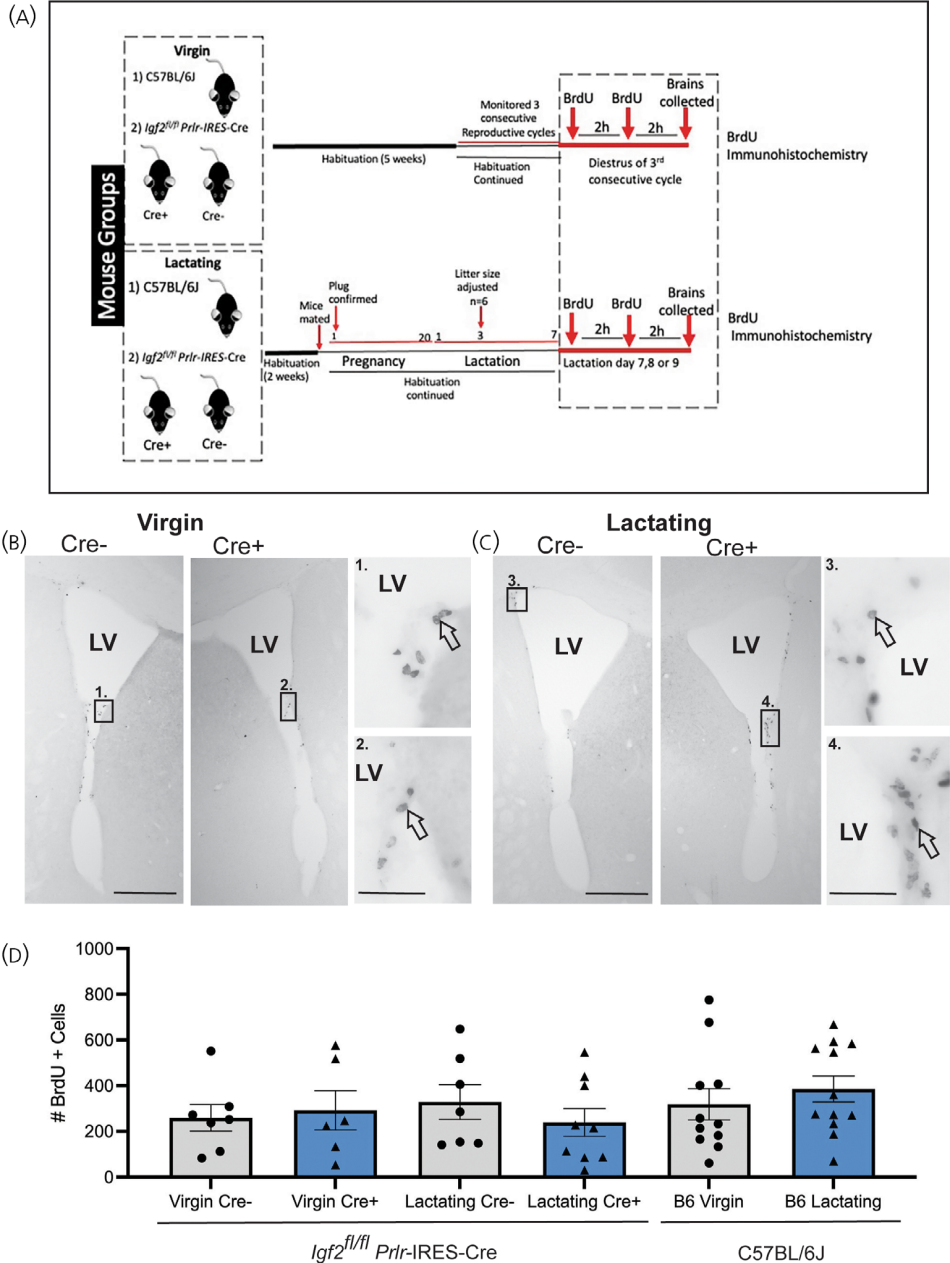
SVZ mitogenesis in virgin and lactating *Igf2* knockout mice. (A) Experimental timeline showing BrdU treatment regime. Images (B/C) show examples of BrdU immunohistochemistry in virgin (B) and lactating (C) *Igf2* ^*fl/fl*^ *Prlr*‐IRES‐Cre mice. Immunopositive cells in the SVZ are depicted by black precipitate and boxed regions 1–4 correspond to higher powered images 1–4. Black arrows point to examples of BrdU positive cells. (D) Graph showing no significant differences in the number of BrdU positive cells labeled throughout the SVZ in both virgin and lactating *Igf2* ^*fl/fl*^ *Prlr*‐IRES‐Cre and C57BL/6J mice. Data are presented as mean ± SEM. LV, Lateral ventricle. Scale bars, 200 μm (B and C), 20 μm (B1, B2, C3, C4).

To evaluate the effect of CP‐derived IGF2 loss on NSC mitogenesis in the SVZ neurogenic niche, groups of virgin and lactating *Igf2* ^*fl/fl*^ *Prlr*‐IRES‐Cre+ and Cre− and C57BL/6J mice were treated with a series of BrdU injections, then brains were collected to assess rates of cell proliferation based on the presence of BrdU immunoreactivity (Figure 5A). No changes in mitogenesis were observed through the SVZ in any of the mouse groups in either virgin or lactating states (Brown–Forsythe and Welch ANOVA, W (5.000, 19.35) = 0.7033, *p* = .6164) (Figure 5B–D).

### 3.5 *Prlr* expression in CP of *Igf2* knockout mouse models

**FIGURE 6:**
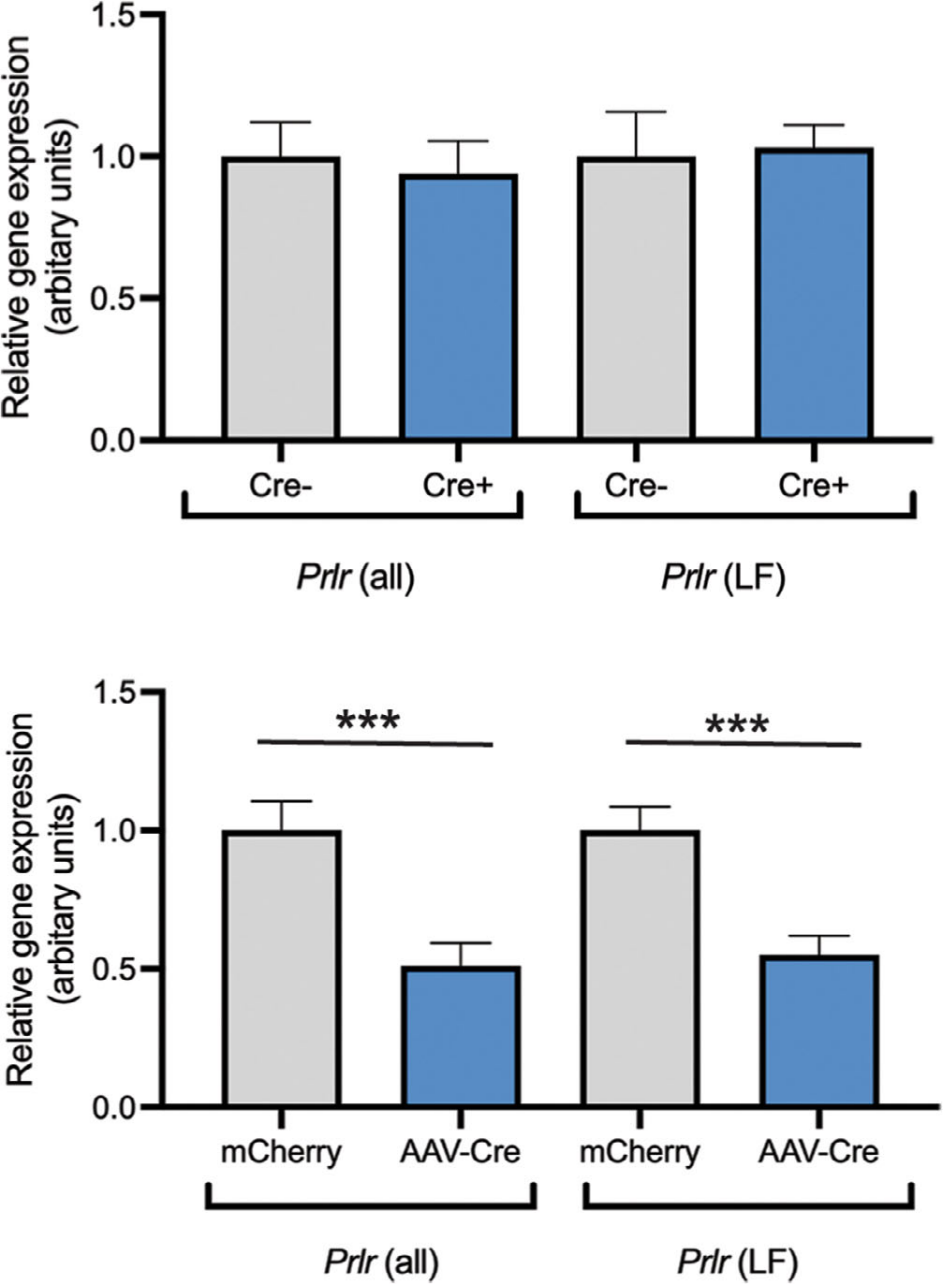
*Prlr* expression in CP tissue from *Igf2* knockout mouse models. No significant differences in the expression of *Prlr* in CP tissue were evident in *Igf2* ^*fl/fl*^ *Prlr*‐IRES‐Cre mice (Top panel, Cre+ *n* = 11, Cre− *n* = 10). *Prlr* expression was reduced approximately 50% in CP tissue collected from *Igf2* ^*fl/fl*^AAV‐Cre mice as compared to controls (Bottom panel, *n* = 10 each, *p* < .0010 (*Prlr* all), *p* < .0006 (*Prlr* LF), unpaired two‐tailed *t* test). Data presented as changes in relative gene expression with respect to the control groups. *Prlr* all detects all forms of the prolactin receptor; *Prlr* LF detects the long form of the prolactin receptor only.

*Prlr* expression in the CP varies between the dark and light phase and across reproductive states with elevated expression levels during lactation.^6^, ^34^ To further investigate the relationship between prolactin and IGF2, we performed qPCR for *Prlr* expression in CP tissue from our *Igf2* knockout mouse models. *Igf2* ^*fl/fl*^ *Prlr*‐IRES‐Cre mice showed no differences in *Prlr* expression between genotypes (unpaired two‐tailed *t* test, *t*(19) = 0.3740, *p* = .7125 (*Prlr* all); *t*(19) = 0.1712, *p* = .8659 (*Prlr* LF), Figure 6A). *Prlr* expression in CP tissue in *Igf2* ^*fl/fl*^ AAV‐Cre mice, however, was around 50% reduced as compared to levels in *Igf2* ^*fl/fl*^ mCherry mice (unpaired two‐tailed *t* test, *t*(18) = 3.708, *p* = .0016 (*Prlr* all); *t*(18) = 4.4145, *p* = .0006 (*Prlr* LF), Figure 6B).

## 4 DISCUSSION

IGF2 is implicated in the pathophysiology of a number of mental illnesses, including anxiety and depression.^35^, ^36^ Hence, this study investigated the hypothesis that prolactin‐induced *Igf2* expression in CP tissue during pregnancy and lactation is required for increases in SVZ mitogenesis necessary to alleviate post‐partum anxiety/depression. IGF2's role in body weight regulation proved problematic in an earlier study generating an adult specific global *Igf2* knockout mouse,^21^ due to the rapid development of a lethal weight loss phenotype. Hence, this study has taken a more targeted approach. Two *Igf2* conditional knockout mouse models have been developed to investigate the specific role of IGF2 derived from CP tissue in the SVZ neurogenic niche. Since *Prlr* are extensively expressed in epithelial cells of the CP^6^, ^23^ (Figure 1C), and we have previously shown prolactin‐induced *Igf2* expression in this tissue,^6^ our first model focused on conditional removal of *Igf2* from all *Prlr*‐containing cells (Figures 2 and S1). For our second model we have utilized an intracerebroventricular injection of an AAV vector to deliver Cre recombinase to epithelial cells of the CP in *Igf2* ^*fl/fl*^ mice (Figure 2). The AAV5 serotype was used as earlier studies have shown it displays tropism preferentially for CP epithelial cells and produces robust expression.^37^, ^38^ Considerable knockdown of *Igf2* in CP tissue was achieved in both of our conditional *Igf2* knockout mouse models (Figure 2).

Mice in which *Igf2* was conditionally removed from *Prlr*‐containing cells (*Igf2* ^*fl/fl*^ *Prlr*‐IRES‐Cre) showed a reduced body weight phenotype in the virgin state (Figure 2D), which was not evident in virgin mice over a 4 week period post‐surgery with AAV‐mediated *Igf2* removal in the CP (*Igf2* ^*fl/fl*^ AAV‐Cre, Figure 2E). This could be due to the apparent developmental timing difference (congenic versus adult onset) of *Igf2* removal or more likely, due to loss of IGF2 in other *Prlr*‐containing tissues. Interestingly, in mice, postnatal expression of *Igf2* declines rapidly in many tissues simultaneously and by 4 weeks expression is diminished.^39^ One of the few regions in adults where production of IGF2 is maintained is in the intestinal stem cell niche.^21^ *Prlr* has been detected throughout the gastrointestinal tract^25^ and thus, mice with *Prlr*‐IRES Cre‐induced deletion of *Igf2* will likely have lost *Igf2* in the gut. Unlike the lethal weight phenotype observed in the previous study using an adult‐specific global *Igf2* knockout mouse,^21^ effects on body weight were minimal in our model. This is perhaps due to *Prlr* expression being limited to a subset of crypts within the colon,^25^ meaning some intact *Igf2* expression would remain elsewhere in the colonic mucosa of these mice. In contrast to the weight loss phenotype observed in our model, investigation of reduced *Igf2* expression in the mouse brain through genetic deletion of the putative enhancer sequence required for brain‐specific *Igf2* expression revealed a link between reduced brain‐specific expression of IGF2 and increased fat deposition.^40^ This effect must be mediated by brain regions independent of the CP, as we did not observe such an effect in either of our models.

IGF2 is expressed in the placenta, and it has been shown that *Igf2* deficiency in placental endocrine cells can cause significant maternal metabolic changes, including failure of pancreatic beta cell mass expansion and alterations in circulating lipids.^41^ In addition, key hormone levels are also affected through decreased prolactin gene expression and impaired *Cyp17a1* expression leading to disruption in estradiol/progesterone ratios.^41^ This work has revealed IGF2—derived from the placenta is—a critical regulator of maternal physiological adaptations to pregnancy.^41^ In our experiments, body weights for both *Igf2* knockout models during pregnancy were not significantly different from controls. Similarly, during lactation, maternal body weight and litter weights were similar across *Igf2* knockout model genotypes, indicative of successful milk production and maternal care. These data suggest that CP IGF2 is not required for the metabolic adaptations seen in pregnancy and lactation.

Manipulating prolactin levels early in pregnancy has been reported to lead to changes in mood during the post‐partum period via upregulation of mitogenesis in the SVZ neurogenic niche, leading to increased numbers of neuroblasts being recruited into olfactory circuitry.^11^ Similarly, elevated prolactin in lactation has been reported to increase mitogenesis in the SVZ.^10^ On the basis of those studies, a key hypothesis underlying the present work was that prolactin's effect on post‐partum mood could be elicited by enhanced neurogenesis initiated by a prolactin‐induced increase in IGF2 secretion from the CP. This hypothesis was not supported by the present data, which rather suggest that CP‐derived IGF2 does not affect SVZ mitogenesis during lactation or anxiety levels in our mouse models post‐partum, when assessed in both the EPM and light/dark transition test (Figure 4). Our results also do not support an earlier study that infused recombinant IGF2 into the lateral ventricle and recorded a consequential increase in slowly proliferating NSCs in the SVZ.^9^ It is important to note here that this earlier study was infusing IGF2 at 100 μg ml^−1^ into the lateral ventricle, which is significantly higher than the physiological IGF2 levels we have measured in CSF across reproductive states (Figure 1E). Our results suggest that the endothelial source of IGF2 within the SVZ neurogenic niche^5^ is either sufficient to regulate NSC proliferation rates or can act in a compensatory capacity to account for the loss of CP‐derived IGF2 post‐partum. Alternatively, it is possible that there are regionally distinct subpopulations responding to IGF2 that we did not detect in our study. A recent study found that changes in physiological states including hunger, satiety, and pregnancy all induce increased activation of spatially distinct NSC sub‐populations in the SVZ rather than a uniform widespread elevation in the rate of mitogenesis.^42^, ^43^ Such an effect may also occur during lactation and would require much more intensive sampling to detect and characterize.

Despite finding no link between loss of CP‐derived IGF2 and anxiety‐like behavior in the post‐partum period, our data establish IGF2 derived from the CP as a critical regulator of olfaction in virgin mice (Figure 3). Unfortunately, this test was not repeated during lactation as olfactory memory from the virgin state may have led to alterations in behavior.^44^ This result, however, corroborates and provides critical insight to earlier findings showing olfactory deficits when Tamoxifen‐inducible virgin adult global *Igf2* knockout mice were assessed using the same buried food test.^21^ Interestingly, we have shown relatively stable IGF2 levels in CSF in both virgin and pregnant states, but significantly higher levels during lactation (Figure 1E), indicative of IGF2 having either more diverse functions or an increasingly critical role at this time. In rodents, lactation is a period associated with enhanced olfactory functions and reduced anxiety to facilitate maternal–pup interactions, including pup recognition and pup retrieval.^28^, ^43^, ^45^, ^46^ Previously, a study in mice investigating olfactory memory processing has shown that mothers show increased recognition of odors after just one exposure to a novel odor as compared to virgin controls.^47^ Olfactory processing initially occurs in the olfactory bulb, the terminal migration site for neuroblasts originating from the SVZ neurogenic niche. Hence, this suggests that elevated IGF2 in CSF during lactation may be contributing to enhanced olfaction at this time. At present, the mechanism for this is poorly understood. Recently, transient waves of temporary interneurons becoming incorporated into the olfactory bulb during pregnancy have been observed and are thought to facilitate maternal care behaviors, such as allowing mothers to recognize their own pups through smell.^43^ Additionally, increased dendritic complexity of newly formed neurons in the olfactory bulb has been attributed as potentially leading to enhanced olfactory function during motherhood.^47^

Multiple studies have observed prolactin‐induced *Igf2* expression in different murine tissues, including mammary gland and CP.^6^, ^48^, ^49^ Interestingly, our data have revealed a 50% reduction in *Prlr* expression following AAV‐mediated deletion of *Igf2* in CP, which did not occur when *Igf2* was conditionally removed from *Prlr*‐containing cells (Figure 6). This suggests that IGF2 may act as a potential regulator of *Prlr* in the CP. It is unclear as to why this reciprocal regulation is only evidenced in one of our mouse models and may be indicative of an adverse effect of AAV and/or high levels of cre recombinase. Alternatively, a compensatory mechanism may have been switched on during development in our mice with conditional *Igf2* knockout in *Prlr*‐containing cells to maintain *Prlr* expression due to much earlier loss of *Igf2*. Despite this, the significance and functional consequences of this reciprocal regulation between *Prlr* and *Igf2* remain to be elucidated.

In summary, we have successfully removed *Igf2* from CP tissue in two different mouse models. Contrary to our original hypothesis, using these models, we found that CP‐derived IGF2 was not necessary to sustain mood in the post‐partum period. We report, however, that IGF2 derived from CP tissue plays a critical role in olfaction, and since previous work has observed improved olfaction during lactation,^47^ we suggest the high IGF2 levels in CSF during lactation measured in our study may be contributing to this. The mechanism for the IGF2‐induced improvement in olfactory function is still unclear, since we observed no changes in the rate of SVZ mitogenesis during lactation or virgin states in mice with conditional *Igf2* knockout. These data suggest that IGF2 may be acting outside of the SVZ neurogenic niche. It is possible that IGF2 travels in CSF through the olfactory CSF conduit^50^ into the olfactory bulb and acts directly either on olfactory mucosa or recently integrated neurons via one or both of its receptors (insulin‐like growth factor 1 receptor (Igf1R) and insulin receptor (IR)), which have been detected in this region.^51^, ^52^ This could promote dendritic outgrowth on newly integrated neurons, as has been previously suggested,^47^ leading to improved olfaction; a critically important maternal adaptation to facilitate appropriate maternal care, which requires further investigation.

## AUTHOR CONTRIBUTIONS

**Hollian R. Phillipps:** Conceptualization; data curation; formal analysis; funding acquisition; investigation; methodology; project administration; resources; validation; visualization; writing – original draft; writing – review and editing. **Eleni C. R. Hackwell:** Investigation; writing – review and editing. **Ionel Sandovici:** Resources; writing – review and editing. **Miguel Constância:** Resources; writing – review and editing. **David R. Grattan:** Conceptualization; funding acquisition; project administration; resources; writing – review and editing.

## FUNDING INFORMATION

This work was funded by a grant from the Neurological Foundation of New Zealand (1943 PG). Miguel Constância was funded by a Medical Research Council grant (MRC_MC_UU_12012/4).

## CONFLICT OF INTEREST STATEMENT

The authors declare no conflicts of interest.

## Supporting information


**Figure S1.** Immunohistochemistry for IGF2 expression in the choroid plexus from mice with either one or two *Igf2* alleles conditionally removed from *Prlr*‐containing cells. IGF2 immunopositive cells (black, examples indicated by red arrows) are evident in mice with single deletion of the *Igf2* allele (maternal or paternal, B) and control mice with both *Igf2* alleles intact (A). Decreased IGF2 expression is evident in mice with homozygous deletion of the two floxed alleles. CSF, cerebrospinal fluid, CP, choroid plexus. Scale bars 50 μm.

**Figure S2.** Additional behavioral parameters measured in the EPM and Light/dark transition test. Graphs (A/B) show the number of times *Igf2* ^*fl/fl*^ *Prlr*‐IRES‐Cre (A) and *Igf2* ^*fl/fl*^ (AAV‐Cre/mCherry) (B) mice entered the open arms over a 5‐min period. Graphs (C/D) provide a measure of the length of time spent on the open arms over the 5‐min testing period for *Igf2* ^*fl/fl*^ *Prlr*‐IRES‐Cre (C) and *Igf2* ^*fl/fl*^ (AAV‐Cre/mCherry) (D) mice. Graphs (E–H) provide record of the distance traveled in the open arms only (E/F) and the total distance covered throughout the EPM (G/H) for *Igf2* ^*fl/fl*^ *Prlr*‐IRES‐Cre (E/G) and *Igf2* ^*fl/fl*^ (AAV‐Cre/mCherry) (F/H) mice. Graphs (I/J) show the number of times *Igf2* ^*fl/fl*^ *Prlr*‐IRES‐Cre (I) and *Igf2* ^*fl/fl*^ (AAV‐Cre/mCherry) (J) mice entered the light chamber. Graphs (K/L) provide a record of the total time *Igf2* ^*fl/fl*^ *Prlr*‐IRES‐Cre+/− (K) and *Igf2* ^*fl/fl*^ (AAV‐Cre/mCherry) (L) mice spend in the light chamber. Unpaired *t*‐tests were used to assess differences between knockout and control groups and data are presented as mean ± SEM. LC, light chamber.

## Data Availability

The original contributions described in the study are included in the manuscript. Further questions can be directed to the corresponding author.

## References

[1] Lehtinen MK , Bjornsson CS , Dymecki SM , Gilbertson RJ , Holtzman DM , Monuki ES . The choroid plexus and cerebrospinal fluid: emerging roles in development, disease, and therapy. J Neurosci. 2013;33(45):17553‐17559. 24198345 10.1523/JNEUROSCI.3258-13.2013 PMC3818536

[2] Shipley FB , Dani N , Xu H , et al. Tracking calcium dynamics and immune surveillance at the choroid plexus blood‐cerebrospinal fluid interface. Neuron. 2020;108(4):623‐639. e10. 32961128 10.1016/j.neuron.2020.08.024 PMC7847245

[3] Falcão AM , Marques F , Novais A , Sousa N , Palha JA , Sousa JC . The path from the choroid plexus to the subventricular zone: go with the flow! Front Cell Neurosci. 2012;6:34. 22907990 10.3389/fncel.2012.00034 PMC3414909

[4] Muhammad T , Wan Y , Sha Q , et al. IGF2 improves the developmental competency and meiotic structure of oocytes from aged mice. Aging (Albany NY). 2021;13(2):2118. 10.18632/aging.202214 PMC7880328 33318299

[5] Ferrón S , Radford E , Domingo‐Muelas A , et al. Differential genomic imprinting regulates paracrine and autocrine roles of IGF2 in mouse adult neurogenesis. Nat Commun. 2015;6(1):8265. 26369386 10.1038/ncomms9265 PMC4579569

[6] Phillipps HR , Rand CJ , Brown RS , Kokay IC , Stanton JA , Grattan DR . Prolactin regulation of insulin‐like growth factor 2 gene expression in the adult mouse choroid plexus. FASEB J. 2019;33(5):6115‐6128. 30735445 10.1096/fj.201802262R

[7] Bracko O , Singer T , Aigner S , et al. Gene expression profiling of neural stem cells and their neuronal progeny reveals IGF2 as a regulator of adult hippocampal neurogenesis. J Neurosci. 2012;32(10):3376‐3387. 22399759 10.1523/JNEUROSCI.4248-11.2012 PMC3338187

[8] DeChiara TM , Robertson EJ , Efstratiadis A . Parental imprinting of the mouse insulin‐like growth factor II gene. Cell. 1991;64(4):849‐859. 1997210 10.1016/0092-8674(91)90513-x

[9] Lozano‐Ureña A , Lázaro‐Carot L , Jiménez‐Villalba E , et al. IGF2 interacts with the imprinted gene Cdkn1c to promote terminal differentiation of neural stem cells. Development. 2023;150(1):dev200563. 36633189 10.1242/dev.200563 PMC9903205

[10] Shingo T , Gregg C , Enwere E , et al. Pregnancy‐stimulated neurogenesis in the adult female forebrain mediated by prolactin. Science. 2003;299(5603):117‐120. 12511652 10.1126/science.1076647

[11] Larsen CM , Grattan DR . Prolactin‐induced mitogenesis in the subventricular zone of the maternal brain during early pregnancy is essential for normal postpartum behavioral responses in the mother. Endocrinology. 2010;151(8):3805‐3814. 20484459 10.1210/en.2009-1385

[12] Phillipps HR , Yip SH , Grattan DR . Patterns of prolactin secretion. Mol Cell Endocrinol. 2020;502:110679. 31843563 10.1016/j.mce.2019.110679

[13] Brunton PJ , Russell JA . The expectant brain: adapting for motherhood. Nat Rev Neurosci. 2008;9(1):11‐25. 18073776 10.1038/nrn2280

[14] Lois C , Alvarez‐Buylla A . Proliferating subventricular zone cells in the adult mammalian forebrain can differentiate into neurons and glia. Proc Natl Acad Sci. 1993;90(5):2074‐2077. 8446631 10.1073/pnas.90.5.2074 PMC46023

[15] Lim DA , Alvarez‐Buylla A . The adult ventricular–subventricular zone (V‐SVZ) and olfactory bulb (OB) neurogenesis. Cold Spring Harb Perspect Biol. 2016;8(5):a018820. 27048191 10.1101/cshperspect.a018820 PMC4852803

[16] Kouremenou I , Piper M , Zalucki O . Adult neurogenesis in the olfactory system: improving performance for difficult discrimination tasks? Bioessays. 2020;42(10):2000065. 10.1002/bies.202000065 32767425

[17] Whitman MC , Greer CA . Adult neurogenesis and the olfactory system. Prog Neurobiol. 2009;89(2):162‐175. 19615423 10.1016/j.pneurobio.2009.07.003 PMC2748178

[18] Leuner B , Sabihi S . The birth of new neurons in the maternal brain: hormonal regulation and functional implications. Front Neuroendocrinol. 2016;41:99‐113. 26969795 10.1016/j.yfrne.2016.02.004 PMC4942360

[19] Deng W , Saxe MD , Gallina IS , Gage FH . Adult‐born hippocampal dentate granule cells undergoing maturation modulate learning and memory in the brain. J Neurosci. 2009;29(43):13532‐13542. 19864566 10.1523/JNEUROSCI.3362-09.2009 PMC2787190

[20] Kuhn HG , Winkler J , Kempermann G , Thal LJ , Gage FH . Epidermal growth factor and fibroblast growth factor‐2 have different effects on neural progenitors in the adult rat brain. J Neurosci. 1997;17(15):5820‐5829. 9221780 10.1523/JNEUROSCI.17-15-05820.1997 PMC6573198

[21] Ziegler AN , Feng Q , Chidambaram S , et al. Insulin‐like growth factor II: an essential adult stem cell niche constituent in brain and intestine. Stem Cell Reports. 2019;12(4):816‐830. 30905741 10.1016/j.stemcr.2019.02.011 PMC6450461

[22] Hammerle CM , Sandovici I , Brierley GV , et al. Mesenchyme‐derived IGF2 is a major paracrine regulator of pancreatic growth and function. PLoS Genet. 2020;16(10):e1009069. 33057429 10.1371/journal.pgen.1009069 PMC7678979

[23] Kokay IC , Wyatt A , Phillipps HR , et al. Analysis of prolactin receptor expression in the murine brain using a novel prolactin receptor reporter mouse. J Neuroendocrinol. 2018;30(9):e12634. 30040149 10.1111/jne.12634

[24] Liu L , Duff K . A technique for serial collection of cerebrospinal fluid from the cisterna magna in mouse. JoVE J Vis Exp. 2008;21:e960. 10.3791/960 PMC2762909 19066529

[25] Aoki M , Wartenberg P , Grünewald R , et al. Widespread cell‐specific prolactin receptor expression in multiple murine organs. Endocrinology. 2019;160(11):2587‐2599. 31373638 10.1210/en.2019-00234

[26] Yang M , Crawley JN . Simple behavioral assessment of mouse olfaction. Curr Protoc Neurosci. 2009;48(1):8.24.1‐8.24.12. 10.1002/0471142301.ns0824s48 PMC2753229 19575474

[27] Walf AA , Frye CA . The use of the elevated plus maze as an assay of anxiety‐related behavior in rodents. Nat Protoc. 2007;2(2):322‐328. 17406592 10.1038/nprot.2007.44 PMC3623971

[28] Maestripieri D , D'Amato FR . Anxiety and maternal aggression in house mice (Mus musculus): a look at interindividual variability. J Comp Psychol. 1991;105(3):295‐301. 1935008 10.1037/0735-7036.105.3.295

[29] Larsen CM , Grattan DR . Exposure to female pheromones during pregnancy causes postpartum anxiety in mice. Vitam Hormones. 2010;83:137‐149. 10.1016/S0083-6729(10)83005-5 20831944

[30] Takao K , Miyakawa T . Light/dark transition test for mice. JoVE J Vis Exp. 2006;1:e104. 10.3791/104 PMC2504462 18704188

[31] Phillipps HR , Ladyman SR , Grattan DR . Maintained expression of genes associated with metabolism in the ventromedial hypothalamic nucleus despite development of leptin resistance during pregnancy in the rat. Physiol Rep. 2013;1(6):e00162. 24400163 10.1002/phy2.162 PMC3871476

[32] Haley VL , Barnes DJ , Sandovici I , et al. Igf2 pathway dependency of the Trp53 developmental and tumour phenotypes. EMBO Mol Med. 2012;4(8):705‐718. 22674894 10.1002/emmm.201101105 PMC3494071

[33] Candlish M , Angelis RD , Götz V , Boehm U . Gene targeting in neuroendocrinology. Compr Physiol. 2011;5(4):1645‐1676. 10.1002/cphy.c140079 26426463

[34] Edelbo BL , Andreassen SN , Steffensen AB , MacAulay N . Day–night fluctuations in choroid plexus transcriptomics and cerebrospinal fluid metabolomics. PNAS Nexus. 2023;2(8):pgad262. 37614671 10.1093/pnasnexus/pgad262 PMC10443925

[35] Mansell T , Novakovic B , Meyer B , et al. The effects of maternal anxiety during pregnancy on IGF2/H19 methylation in cord blood. Transl Psychiatry. 2016;6(3):e765. 27023171 10.1038/tp.2016.32 PMC4872456

[36] Luo Y‐W , Xu Y , Cao W‐Y , et al. Insulin‐like growth factor 2 mitigates depressive behavior in a rat model of chronic stress. Neuropharmacology. 2015;89:318‐324. 25446675 10.1016/j.neuropharm.2014.10.011

[37] Jang A , Lehtinen MK . Experimental approaches for manipulating choroid plexus epithelial cells. Fluids and Barriers of the CNS. 2022;19(1):36. 35619113 10.1186/s12987-022-00330-2 PMC9134666

[38] Mazucanti CH , Kennedy V Jr , Premathilake HU , et al. AAV5‐mediated manipulation of insulin expression in choroid plexus has long‐term metabolic and behavioral consequences. Cell Rep. 2023;42(8):112903. 37515772 10.1016/j.celrep.2023.112903 PMC10529429

[39] Lui JC , Finkielstain GP , Barnes KM , Baron J . An imprinted gene network that controls mammalian somatic growth is down‐regulated during postnatal growth deceleration in multiple organs. Am J Physiol Regul Integr Comp Physiol. 2008;295(1):R189‐R196. 18448610 10.1152/ajpregu.00182.2008 PMC2494817

[40] Jones BK , Levorse J , Tilghman SM . Deletion of a nuclease‐sensitive region between the Igf2 and H19 genes leads to Igf2 misregulation and increased adiposity. Hum Mol Genet. 2001;10(8):807‐814. 11285246 10.1093/hmg/10.8.807

[41] Lopez‐Tello J , Yong HE , Sandovici I , et al. Fetal manipulation of maternal metabolism is a critical function of the imprinted Igf2 gene. Cell Metab. 2023;35(7):1195‐1208. e6. 37437545 10.1016/j.cmet.2023.06.007 PMC7619417

[42] Paul A , Chaker Z , Doetsch F . Hypothalamic regulation of regionally distinct adult neural stem cells and neurogenesis. Science. 2017;356(6345):1383‐1386. 28619719 10.1126/science.aal3839

[43] Chaker Z , Segalada C , Kretz JA , et al. Pregnancy‐responsive pools of adult neural stem cells for transient neurogenesis in mothers. Science. 2023;382(6673):958‐963. 37995223 10.1126/science.abo5199

[44] Aqrabawi AJ , Kim JC . Behavioral evaluation of odor memory in mice. Bio‐protocol. 2018;8(18):e3023. 34395811 10.21769/BioProtoc.3023 PMC8328584

[45] Smotherman WP , Bell RW , Starzec J , Elias J , Zachman TA . Maternal responses to infant vocalizations and olfactory cues in rats and mice. Behav Biol. 1974;12(1):55‐66. 4429513 10.1016/s0091-6773(74)91026-8

[46] Gandelman R , Zarrow M , Denenberg VH . Maternal behavior: differences between mother and virgin mice as a function of the testing procedure. Dev Psychobiol. 1970;3(3):207‐214. 5527422 10.1002/dev.420030308

[47] Belnoue L , Malvaut S , Ladevèze E , Abrous DN , Koehl M . Plasticity in the olfactory bulb of the maternal mouse is prevented by gestational stress. Sci Rep. 2016;6(1):37615. 27886228 10.1038/srep37615 PMC5122868

[48] Carver KC , Schuler LA . Prolactin does not require insulin‐like growth factor intermediates but synergizes with insulin‐like growth factor I in human breast cancer cells. Mol Cancer Res. 2008;6(4):634‐643. 18403642 10.1158/1541-7786.MCR-07-2069

[49] Brisken C , Ayyannan A , Nguyen C , et al. IGF‐2 is a mediator of prolactin‐induced morphogenesis in the breast. Dev Cell. 2002;3(6):877‐887. 12479812 10.1016/s1534-5807(02)00365-9

[50] Ethell DW . Disruption of cerebrospinal fluid flow through the olfactory system may contribute to Alzheimer's disease pathogenesis. J Alzheimers Dis. 2014;41(4):1021‐1030. 24769627 10.3233/JAD-130659

[51] McCurdy RD , Féron F , McGrath JJ , Mackay‐Sim A . Regulation of adult olfactory neurogenesis by insulin‐like growth factor‐I. Eur J Neurosci. 2005;22(7):1581‐1588. 16197498 10.1111/j.1460-9568.2005.04355.x

[52] Lacroix MC , Badonnel K , Meunier N , et al. Expression of insulin system in the olfactory epithelium: first approaches to its role and regulation. J Neuroendocrinol. 2008;20(10):1176‐1190. 18752648 10.1111/j.1365-2826.2008.01777.x

