# Supplementary figures and images for: Loss of choroid plexus‐derived insulin‐like growth factor 2 (IGF2) leads to hyposmia, while retaining post‐partum mood resilience in mice
Source: J Neuroendocrinol. 2025 Jun 3;37(9):e70058. doi: 10.1111/jne.70058 (PMC12404905; doi:10.1111/jne.70058)

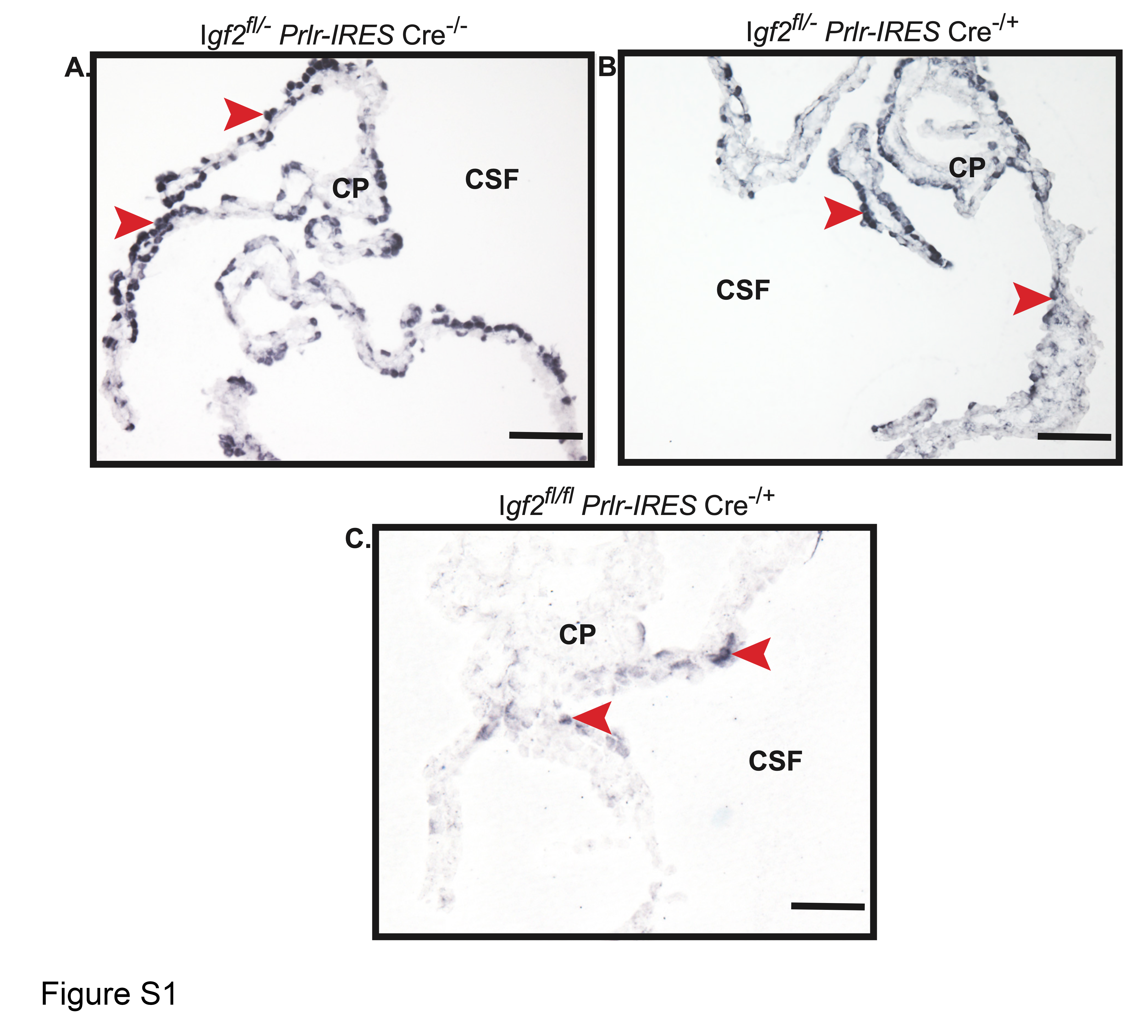

Supplement: Supplementary file 1 — Figure S1. Immunohistochemistry for IGF2 expression in the choroid plexus from mice with either one or two Igf2 alleles conditionally removed from Prlr‐containing cells. IGF2 immunopositive cells (black, examples indicated by red arrows) are evident in mice with single deletion of the Igf2 allele (maternal or paternal, B) and control mice with both Igf2 alleles intact (A). Decreased IGF2 expression is evident in mice with homozygous deletion of the two floxed alleles. CSF, cerebrospinal fluid, CP, choroid plexus. Scale bars 50 μm. [file JNE-37-e70058-s001.tif]

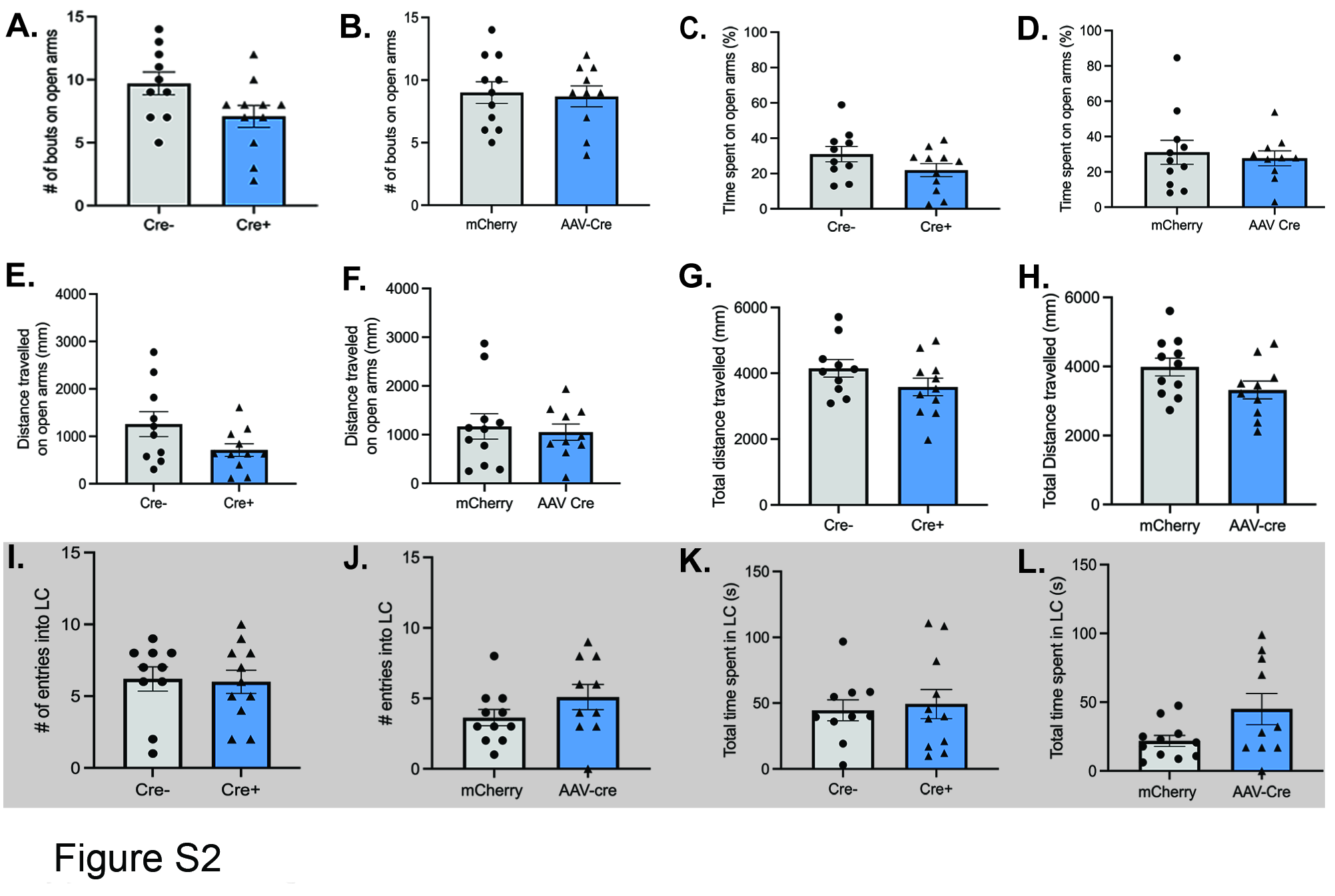

Supplement: Supplementary file 2 — Figure S2. Additional behavioral parameters measured in the EPM and Light/dark transition test. Graphs (A/B) show the number of times Igf2 fl/fl Prlr‐IRES‐Cre (A) and Igf2 fl/fl (AAV‐Cre/mCherry) (B) mice entered the open arms over a 5‐min period. Graphs (C/D) provide a measure of the length of time spent on the open arms over the 5‐min testing period for Igf2 fl/fl Prlr‐IRES‐Cre (C) and Igf2 fl/fl (AAV‐Cre/mCherry) (D) mice. Graphs (E–H) provide record of the distance traveled in the open arms only (E/F) and the total distance covered throughout the EPM (G/H) for Igf2 fl/fl Prlr‐IRES‐Cre (E/G) and Igf2 fl/fl (AAV‐Cre/mCherry) (F/H) mice. Graphs (I/J) show the number of times Igf2 fl/fl Prlr‐IRES‐Cre (I) and Igf2 fl/fl (AAV‐Cre/mCherry) (J) mice entered the light chamber. Graphs (K/L) provide a record of the total time Igf2 fl/fl Prlr‐IRES‐Cre+/− (K) and Igf2 fl/fl (AAV‐Cre/mCherry) (L) mice spend in the light chamber. Unpaired t‐tests were used to assess differences between knockout and control groups and data are presented as mean ± SEM. LC, light chamber. [file JNE-37-e70058-s002.tif]
